# Oral vancomycin treatment does not alter markers of postprandial inflammation in lean and obese subjects

**DOI:** 10.14814/phy2.14199

**Published:** 2019-08-18

**Authors:** Guido J. Bakker, Johan G. Schnitzler, Siroon Bekkering, Nicolien C. de Clercq, Annefleur M. Koopen, Annick V. Hartstra, Emma C. E. Meessen, Torsten P. Scheithauer, Maaike Winkelmeijer, Geesje M. Dallinga‐Thie, Patrice D. Cani, Elles Marleen Kemper, Maarten R. Soeters, Jeffrey Kroon, Albert K. Groen, Daniël H. van Raalte, Hilde Herrema, Max Nieuwdorp

**Affiliations:** ^1^ Department of Vascular Medicine Amsterdam UMC, Location AMC at University of Amsterdam Amsterdam The Netherlands; ^2^ Department of Experimental Vascular Medicine Amsterdam UMC, Location AMC at University of Amsterdam Amsterdam The Netherlands; ^3^ Department of Experimental Internal Medicine Radboud University Medical Centre Nijmegen The Netherlands; ^4^ Department of Endocrinology and Metabolism Amsterdam UMC, Location AMC at University of Amsterdam Amsterdam The Netherlands; ^5^ Department of Internal Medicine, Diabetes Center Amsterdam UMC, Location VUMC at Vrije Universiteit Amsterdam Amsterdam The Netherlands; ^6^ WELBIO – Walloon Excellence in Life Sciences and Biotechnology, Metabolism and Nutrition Louvain Drug Research Institute, Université Catholique de Louvain Brussels Belgium; ^7^ Department of Clinical Pharmacy Amsterdam UMC, Location AMC at University of Amsterdam Amsterdam The Netherlands; ^8^ Amsterdam UMC, ICar at Vrije Universiteit Amsterdam Amsterdam The Netherlands; ^9^ Department of Molecular and Clinical Medicine, Wallenberg Laboratory Sahlgrenska Academy, University of Gothenburg Gothenburg Sweden

**Keywords:** Bacterial endotoxins, bacterial translocation, inflammation, obesity

## Abstract

Intake of a high‐fat meal induces a systemic inflammatory response in the postprandial which is augmented in obese subjects. However, the underlying mechanisms of this response have not been fully elucidated. We aimed to assess the effect of gut microbiota modulation on postprandial inflammatory response in lean and obese subjects. Ten lean and ten obese subjects with metabolic syndrome received oral vancomycin 500 mg four times per day for 7 days. Oral high‐fat meal tests (50 g fat/m^2^ body surface area) were performed before and after vancomycin intervention. Gut microbiota composition, leukocyte counts, plasma lipopolysaccharides (LPS), LPS‐binding protein (LBP), IL‐6 and MCP‐1 concentrations and monocyte CCR2 and cytokine expression were determined before and after the high‐fat meal. Oral vancomycin treatment resulted in profound changes in gut microbiota composition and significantly decreased bacterial diversity in both groups (phylogenetic diversity pre‐ versus post‐intervention: lean, 56.9 ± 7.8 vs. 21.4 ± 6.6, *P* < 0.001; obese, 53.9 ± 7.8 vs. 21.0 ± 5.9, *P* < 0.001). After intervention, fasting plasma LPS significantly increased (lean, median [IQR] 0.81 [0.63–1.45] EU/mL vs. 2.23 [1.33–3.83] EU/mL, *P* = 0.017; obese, median [IQR] 0.76 [0.45–1.03] EU/mL vs. 1.44 [1.11–4.24], *P* = 0.014). However, postprandial increases in leukocytes and plasma LPS were unaffected by vancomycin in both groups. Moreover, we found no changes in plasma LBP, IL‐6 and MCP‐1 or in monocyte CCR2 expression. Despite major vancomycin‐induced disruption of the gut microbiota and increased fasting plasma LPS, the postprandial inflammatory phenotype in lean and obese subjects was unaffected in this study.

## Introduction

In the past decade, a plethora of animal and human studies have shown an important link between the composition of the gut microbiota and metabolic diseases, such as obesity and type 2 diabetes mellitus (T2D) (Turnbaugh et al. 2009; Qin et al. 2012; Tremaroli and Backhed 2012; Karlsson et al. 2013). These diseases are characterized by a chronic state of low‐grade systemic inflammation (Chawla et al. 2011; Gregor and Hotamisligil 2011). Translocation of metabolites derived from intestinal bacteria into the systemic circulation has been suggested to underlie the pathophysiology of this inflammatory state (Cani et al. 2007; Cani et al. 2008). For example, mice fed a high‐fat diet for 4 weeks had increased amounts of bacterial DNA in both circulating blood and mesenteric visceral adipose tissue (Amar et al. 2011). In humans, plasma concentrations of lipopolysaccharide (LPS, also referred to as endotoxin), a cell wall component of Gram‐negative bacteria, have been associated with obesity (Basu et al. 2011), T2D (Pussinen et al. 2011) and cardiovascular disease (Pussinen et al. 2007). Similarly, plasma LPS‐binding protein (LBP), a marker of LPS exposure, has been associated with obesity and insulin resistance (Sun et al. 2010; Moreno‐Navarrete et al. 2012). Translocation of intestinal LPS into the blood is actively mediated by enterocytes through the apical scavenger receptor class B type 1 (SR‐BI), which binds LPS and mediates incorporation of LPS in chylomicrons (Vishnyakova et al. 2003; Beaslas et al. 2009). Thus, endotoxemia may especially occur after a meal enriched with fat.

Indeed, several human studies have shown after administration of a high‐fat meal, a postprandial increase in plasma LPS (Erridge et al. 2007; Ghanim et al. 2009; Laugerette et al. 2011), accompanied by a systemic increase in IL‐6 (Blackburn et al. 2006; Lundman et al. 2007; Khoury et al. 2010; Laugerette et al. 2011) and TNF‐*α* (Nappo et al. 2002) and a shift towards a proinflammatory phenotype of circulating monocytes (Gower et al. 2011). This postprandial inflammatory response was found to be increased in obesity and T2D with the transient postprandial rise in LPS being significantly higher in obese (Clemente‐Postigo et al. 2012; Vors et al. 2015) and T2D (Harte et al. 2012) individuals compared to lean participants. Moreover, consumption of a high‐fat meal increased plasma IL‐6 and TNF‐*α* concentrations to a greater extent in T2D compared to healthy subjects (Nappo et al. 2002). Notably, in these trials endotoxin concentrations were closely related to postprandial plasma chylomicrons (CMs), further suggesting postprandial cotransport of LPS with dietary lipids.

As abundance of Gram‐negative bacteria is the main driver of intestinal LPS concentration, we hypothesized that the composition of gut microbiota is an important factor in LPS translocation in the postprandial phase. In order to test this hypothesis, we treated our subjects with oral vancomycin, a broad‐spectrum antibiotic that targets Gram‐positive bacteria, resulting in overgrowth of Gram‐negative strains and increasing luminal LPS concentrations. In this study, we aimed to assess the effect of oral vancomycin on postprandial LPS translocation and the inflammatory response in lean subjects and in obese subjects who fulfil the metabolic syndrome criteria.

## Methods

### Study design

Lean (body mass index [BMI] between 18.5 and 25 kg/m^2^) and obese (BMI ≥ 30 kg/m^2^), metabolic syndrome (≥3/6 criteria: waist circumference >102 cm; blood pressure ≥130 mmHg systolic or ≥85 mmHg diastolic; fasting plasma glucose ≥5.6 mmol/L; high‐density lipoprotein cholesterol [HDL‐C] <1.03 mmol/L; fasting triglycerides ≥1.7 mmol/L; homeostatic model assessment‐insulin resistance [HOMA‐IR] >2.2) male subjects aged 18–75 years were recruited via local advertisements. Subjects were excluded if they met one of the following criteria: use of any medication; use of antibiotics or proton pump inhibitors in the past 3 months; a medical history of type 1 or 2 diabetes, stroke, myocardial infarction, pacemaker, or cholecystectomy; smoking; use of >5 units of alcohol daily; recreational drug use; use of pre‐, pro‐ or synbiotics. All participants received oral vancomycin 500 mg four times a day for 7 days. High‐fat meal tests were scheduled before and after vancomycin intervention. To ensure a complete wash‐out of the study medication, postintervention visits were scheduled two days after cessation of vancomycin. Subjects were asked to refrain from strenuous exercise during the study and to keep 3‐day online food diaries in the days preceding the study visits. All participants gave written informed consent for participation in the study. The study was approved by the Institutional Review Board of the Amsterdam UMC, location AMC in Amsterdam, the Netherlands, and conducted in accordance with the Declaration of Helsinki (version 2013).

### High‐fat meal tests

Subjects visited the hospital after an overnight fast of at least 10 h. After measurement of height, weight, waist and hip circumference, and blood pressure, a cannula was inserted into the antecubital vein for blood sampling. Blood samples were collected before (*t* = 0 h) and after (*t* = 2 h and *t* = 4 h) ingestion of the liquid high‐fat meal (fresh cream, 35% fat, Albert Heijn, Zaandam, the Netherlands, 93 E% fat, 4 E% carbohydrate, 3 E% protein) containing 335 kcal, 35 g fat of which 23 g saturated and 12 g mono‐unsaturated, 3.0 g carbohydrate of which 3.0 g sugars, and 2.5 g protein per 100 mL in a dosage of 50 g of fat per square meter body surface area (BSA) (Klop et al. 2013). BSA was calculated using the following formula: BSA (*m* ^2^) = √((height in cm × weight in kg)/3600) (Mosteller 1987). During the high‐fat meal tests, subjects were not allowed to eat or drink, except water, and refrained from physical activity.

### Biochemical analyses

Blood was collected in Vacutainer® tubes containing heparin, Ethylenediaminetetraacetic acid (EDTA), or spray‐coated silica and a polymer gel for serum separation (Beckton Dickinson, Franklin Lakes, NJ), centrifuged at 1550 g (15 min, 4°C) and plasma and serum were stored at −80°C until further analyses. Plasma glucose was determined with a commercial assay on a Cobas 8000 c702 analyzer (Roche, Basel, Switzerland). Plasma insulin was determined using the ADVIA Centaur XP Immunoassay System (Siemens, Erlangen, Germany), according to the manufacturer’s protocol. Plasma total cholesterol, HDL‐C, and triglycerides were determined using commercial assays (Diasys and WAKO) on the Selectra^®^ (Sopachem, Ochten, the Netherlands) according to the manufacturer’s instructions. Low‐density lipoprotein cholesterol (LDL‐C) levels were calculated using the Friedewald formula. Apolipoprotein (apo) B and apo A1 were analyzed using a commercial nephelometric assay on a Selectra® autoanalyzer.

Plasma for LPS measurements was transferred and stored using endotoxin‐free materials only. LPS concentrations (i.e., endotoxin activity in EU/mL) were determined using the Endosafe‐MCS (Charles River Laboratories, Lyon, France) based on the Limulus Amebocyte Lysate (LAL) kinetic chromogenic methodology measuring color intensity directly related to the endotoxin activity present in a sample as previously described (Everard et al. 2013). Briefly, plasma was first diluted 1/10 with endotoxin‐free buffer (Charles River Laboratories, Lyon, France) and endotoxin‐free LAL reagent water (Charles River Laboratories, Lyon, France) to minimize interferences in the reaction and then heated for 15 min at 70°C. Each sample was further diluted with LAL water and treated in duplicate. The dilution was chosen in order to fully recover the LPS spikes (>70%). Two spikes for each sample were included in the determination. All samples were validated for the recovery and the coefficient variation. The lower limit of detection was 0.005 EU/mL. Plasma concentrations of IL‐6, MCP‐1, and LBP were determined using commercially available ELISA kits (IL‐6 Human ELISA Kit, High Sensitivity, Thermo Fisher Scientific, Waltham, MA; Human CCL2 (MCP‐1) ELISA Ready‐SET‐Go!®, eBioscience, San Diego, CA; LBP, Human, ELISA kit, Hycult Biotech Inc., Plymouth Meeting, PA, respectively) according to the manufacturer’s instruction. Blood cell counts were determined automatically using an XN‐9000 analyzer (Kobe, Hyōgo Prefecture, Japan) according to the manufacturer’s instructions within 45 min after collection in EDTA‐coated tubes that were kept at room temperature.

### Gut microbiota analyses

Fecal samples were collected by the participants at home, stored at 4°C, transported to the hospital within 24 h and stored at −80°C until further processing. Before collection, subjects received three feces collection tubes (Faeces tubes 76 × 20 mm, Nümbrecht, Germany) and sample collection instruction. Total genomic DNA was isolated from 250 mg of feces using an adapted repeated bead‐beating method (Salonen et al. 2010). 16S genes where amplified with index primers with an adapted PCR method (Kozich et al. 2013). After confirmation of PCR products on agarose gel the samples were purified and equal molar pooled before being analyzed by Illumina Miseq (V3,600) sequencing (Kozich et al. 2013) (see Supplements (https://figshare.com/s/25e137d98f1d736ab5e2)).

To allow interpretation of the sequences, forward and reverse reads were length trimmed at 240 and 210 nucleotides respectively and amplicon sequence variants (ASVs) were inferred and merged using dada2 V1.5.2 (Callahan et al. 2016). Taxonomy was assigned using dada2 implementation of the RDP classifier (Wang et al. 2007) and SILVA (Quast et al. 2013) 16S ribosomal database V128. Microbiota data were further analyzed and visualized using phyloseq (McMurdie and Holmes 2013), vegan (Oksanen et al. 2018) and picante (Kembel et al. 2014). Differences in beta‐diversity were tested with permutational multivariate analysis of variance using weighted UniFrac (Lozupone and Knight 2005). All statistical tests concerning alpha and beta‐diversity were performed on rarefied ASV tables.

To determine the abundance of intact bacteria before and after vancomycin, fecal bacteria were stained with the nucleic acid stain SytoBC (ThermoFisher Scientific, Invitrogen, Carlsbad, CA). Frozen fecal samples (100 mg) were diluted 1:10 in phosphate buffered saline (PBS) with cOmpleteTM Protease Inhibitory Cocktail (Roche, Basel, Switzerland). All steps were performed on ice. Samples were vigorously shaken for 10 min to obtain a homogenized solution which was centrifuged at 400*g* for 5 min to separate the bacteria from large debris. Next, 100 *µ*L of the supernatant was taken and centrifuged for 5 min at 8000*g* to pellet the bacteria. The pellet was washed with 1 mL of PBS. Lastly, bacteria were stained with 1:4000 diluted SytoBC stain in a 0.9% NaCl solution with 0.1 mol/L (4‐(2‐hydroxyethyl)‐1‐piperazineethanesulfonic acid) (HEPES). Stained bacteria were measured on a BD FACS Canto II (Becton, Dickinson, Franklin Lakes, NJ) and analyzed using FlowJo software (version 10.0 FlowJo, LLC, Ashland, OR). Threshold settings were set to the minimal allowable voltage for side scatter to be able to measure small particles. At least 50.000 events were counted. The gating strategy is outlined in Figure S1 (https://figshare.com/s/515d569aff0c63e01a92).

### Monocyte flow cytometry

Seven hundred microliters of whole blood was lysed in 6.3 mL red blood cell lysis buffer (Affymetrix, eBioscience, San Diego, CA) for 15 min. The reaction was stopped by the addition of 28 mL PBS. After centrifugation at 387*g* (5 min, 4°C) the peripheral blood mononuclear cells (PBMCs) were harvested. The PBMCs were suspended in 200 *µ*L PBS and incubated with fluorochrome labeled antibodies (PE‐Cy7, mouse anti‐human CD14, BD Pharmingen, clone: M5E2, ref# 557742, lot# 6273595, 1/50 dilution; APC‐H7, mouse anti‐human CD16, BD Pharmingen, clone: 3G8, ref# 560195, lot# 5061807, 1/50 dilution; PerCP‐Cy5.5, mouse anti‐human HLA‐DR, BD Pharmingen, clone: G46‐6, ref# 560652, lot# 6098644, 1/50 dilution; Alexa Fluor 647, mouse anti‐human CD192 (CCR2), BD Pharmingen, clone: 48607, ref# 558406, lot# 6165900, 1/50 dilution) for 15 min (room temperature, dark). After incubation, the labeled cells were washed with 140 *µ*L PBS and centrifuged at 441*g* (3 min, 4°C). The cell pellet was resuspended in 200 *µ*L PBS. Samples were analyzed on a BD FACS Canto II (Becton, Dickinson, Franklin Lakes, NJ). Five thousand monocyte events were counted per sample. Monocyte subsets (i.e., classical, intermediate, and nonclassical monocytes) were classified according to HLA‐DR, CD14, and CD16 expression (classical: CD14++, CD16–; intermediate: CD14++, CD16+; nonclassical: CD14+, CD16+). The gating strategy is outlined in Figure S2 (https://figshare.com/s/19152c97212b53a10ff7). Within the monocyte subsets the expression of C‐C chemokine receptor type 2 (CCR2) was determined. The delta median fluorescence intensity (DMFI = MFI surface staining – MFI backbone [CD14, CD16, HLA‐DR]) was analyzed using FlowJo software (version 10.0 FlowJo, LLC, Ashland, OR).

### Ex vivo monocyte stimulation

PBMCs were isolated using a LymphoprepTM (Axis‐Shield, Dundee, Scotland) density gradient and subsequent CD14^+^ monocyte isolation was conducted by magnetic activated cell sorting (MACS) using human CD14‐coated MicroBeads according to the manufacturer’s instructions (MACS, Miltenyi Biotec, Leiden, the Netherlands). Next, the monocytes were counted (Casy TT, Roche Innovatis, Basel, Switzerland) and plated into a 96‐well flat‐bottom plate (1E5 monocytes/well), followed by culturing in Roswell Park Memorial Institute (RPMI) + GlutaMAX supplemented with 25 mmol/L HEPES and 1% penicillin/streptomycin (Gibco, Dun Laoghaire, County Dublin, Ireland). Subsequently, monocytes were stimulated for 24 h with either RPMI, 10 ng/mL LPS or 10 *μ*g/mL Pam3Cys at 37°C, 5% CO_2_ after which the supernatants were collected and stored at −20°C. Cytokine production (IL‐1*β*, TNF‐*α*, MCP‐1, IL‐6 and IL‐10) was determined in supernatants using LEGENDplexTM bead‐based immunoassay according to the manufacturer’s instructions (Biolegend, San Diego, CA).

### Statistical analyses

Data were checked for normality with the Shapiro–Wilk test and by visually assessing histograms and normality plots. Effects of vancomycin on fasting parameters were assessed using the paired *t* test for normal continuous variables and the Wilcoxon signed rank test for other variables. One‐way ANOVA for repeated measures (rm‐ANOVA) for normal continuous variables and the Friedman test for other variables, with Bonferroni post hoc testing, was used to assess effects of the high‐fat meal on postprandial parameters. Two‐way repeated measures ANOVA with Bonferroni post hoc testing with time after the meal (time) and treatment (pre vs. post intervention) as factors was used to determine the effect of treatment (time × treatment interaction) on postprandial parameters. Statistical analyses were performed using SPSS Statistics software, version 24 (IBM, Armonk, New York). Data are provided as mean with standard deviation (SD) or median with interquartile range (IQR). *P*‐values < 0.05 were considered statistically significant. All authors had access to the study data and reviewed and approved the final manuscript.

## Results

We included 10 lean and 10 obese Caucasian males. Baseline characteristics are summarized in Table 1. All obese subjects met the metabolic syndrome criteria and were insulin resistant (HOMA‐IR score ≥ 2.2). All subjects completed the study. No side effects of vancomycin treatment were reported, apart from a mild increase in stool frequency in eight subjects (four subjects in each group). Caloric intake and body weight remained unchanged in both groups throughout the study period (data not shown).

**Table 1:** Baseline characteristics.

	Lean (*n* = 10)	Obese (*n* = 10)
Age (years)	28.7 (8.7)	58.5 (7.3)
BMI (kg/m^2^)	22.8 (1.1)	34.6 (4.2)
BSA (m^2^)	1.99 (0.16)	2.41 (0.26)
Waist circumference (cm)	79.5 (6.0)	114.1 (14.5)
Waist/hip ratio	0.92 (0.04)	1.06 (0.06)
Systolic blood pressure (mmHg)	130.8 (10.3)	155.9 (14.7)
Diastolic blood pressure (mmHg)	78.0 (9.9)	93.8 (12.3)
Leukocytes (10E9/L)	5.3 (0.8)	6.0 (1.1)
Fasting glucose (mmol/L)	4.8 (0.2)	6.1 (0.7)
Fasting insulin (pmol/L)*	29 (15–43)	89 (66–128)
HOMA‐IR*	0.9 (0.5–1.4)	3.4 (2.6–4.5)
Dietary intake		
Energy (kcal/day)	2359 (652)	2046 (289)
Fat (g/day)	86.8 (30.9)	74.6 (18.0)
Carbohydrates (g/day)	279.3 (81.4)	218.0 (58.4)
Protein (g/day)	92.8 (31.2)	97.3 (12.8)
Fiber (g/day)	25.1 (9.7)	16.6 (25.1)

Data are represented as mean (SD) unless otherwise specified. BMI, body mass index; BSA, body surface area; HOMA‐IR, homeostasis model assessment of insulin resistance; *n*, number of patients.

* Data are represented as median [IQR].

### Vancomycin significantly alters fecal microbiota composition

**Figure 1:**
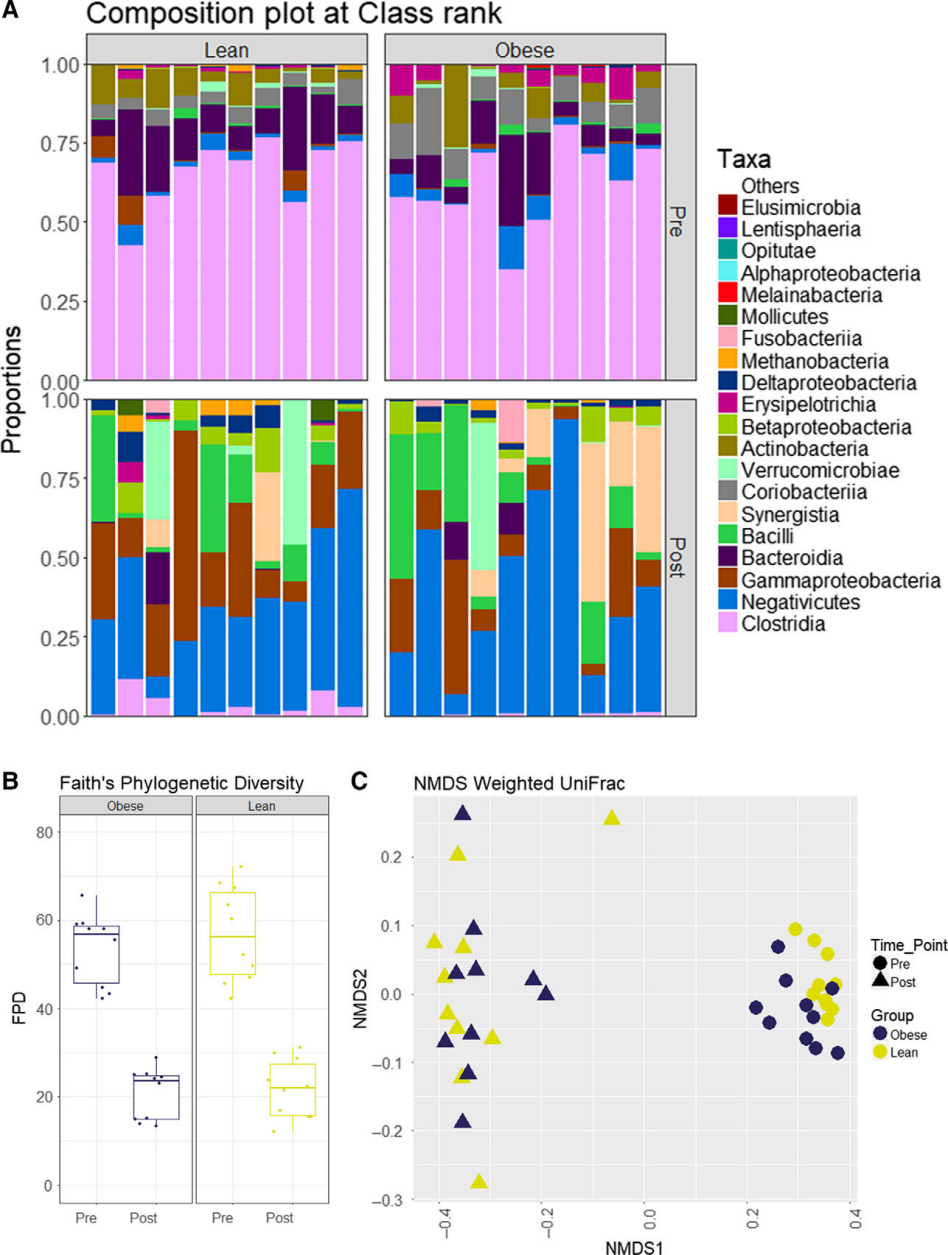
Effect of vancomycin on gut microbiota composition. Fecal microbial community characteristics of obese and lean subjects before and after treatment. Vancomycin treatment resulted in significant changes in gut microbiota composition in both groups. (A) microbial composition showing the 20 most abundant bacterial classes (upper part, preintervention; lower part, postintervention). (B) Faith’s phylogenetic diversity distribution showed a significant decrease in alpha‐diversity (lean, *P* < 0.001; obese, *P* < 0.001). (C) NMDS ordination of the weighted UniFrac distances showed a significant shift in beta‐diversity (lean, *P* = 0.001; obese, *P* = 0.001).

As expected, vancomycin significantly altered the relative abundance of several bacterial groups (Fig. 1 A). Specifically, major Gram‐positive taxa such as Clostridia and Bacteroidia decreased, while Gram‐negative taxa (Negativicutes and Proteobacteria) and vancomycin‐resistant Gram‐positive taxa such as Bacilli increased. Vancomycin decreased bacterial diversity (Faith’s phylogenetic diversity pre and post intervention respectively: lean, 56.9 ± 7.8 vs. 21.4 ± 6.6, *P* < 0.001; obese, 53.9 ± 7.8 vs. 21.0 ± 5.9, *P* < 0.001; Fig. 1B). Non‐metric multidimensional scaling (NMDS) ordination of the weighted UniFrac distances showed a significant shift of gut microbiota composition after treatment (lean, *P* = 0.001; obese, *P* = 0.001; Fig. 1C). The percentage of intact fecal bacteria as measured using SytoBC positivity was also significantly reduced (lean, 82.4 ± 5.8% vs. 38.4 ± 11.4%, *P* < 0.001; obese 76.1 ± 15.7% vs. 35.4 ± 9.0%, *P* < 0.001).

### Vancomycin increases fasting LPS, without significantly affecting the inflammatory state

**Figure 2:**
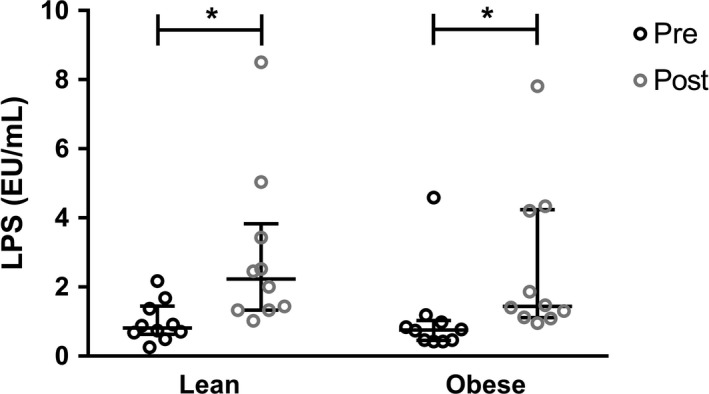
Effect of vancomycin on fasting plasma LPS. Vancomycin treatment significantly increased fasting plasma LPS in both groups (Wilcoxon signed rank test; lean, *P* = 0.017; obese, *P* = 0.014). Lines at median and IQR.

After vancomycin treatment, fasting LPS significantly increased in both groups (lean, median [IQR]: 0.81 [0.63–1.45] versus 2.23 [1.33–3.83], *P* = 0.017; obese, median [IQR]: 0.76 [0.45–1.03] versus 1.44 [1.11–4.24], *P* = 0.014; Fig. 2). However, plasma LBP, IL‐6 and MCP‐1 did not change significantly (Table 2 and Table S1 [https://figshare.com/s/25e137d98f1d736ab5e2]). On a cellular level, there was no effect on leukocyte counts (Table 2), leukocyte subtypes (Table S2 [https://figshare.com/s/25e137d98f1d736ab5e2]) and distribution of monocyte subtypes (i.e. classical, intermediate and nonclassical, Table S3). Monocyte expression of CCR2, which was previously shown to be positively correlated with lipid levels and inflammation (Bernelot Moens et al. 2017; Verweij et al. 2018), did not differ significantly in the fasting state upon vancomycin treatment in the lean subjects but decreased in the obese group (Table 2). Finally, vancomycin treatment resulted in lower monocyte production of MCP‐1 only in the lean group upon *ex vivo* stimulation with LPS (Fig. S3 [https://figshare.com/s/2a0471bb2f08d5c9db56]). IL‐1*β* production was nonsignificantly reduced in Toll‐like receptor 2 (TLR2)‐stimulated monocytes after treatment compared to baseline in both the lean and obese subjects. Production of other cytokines (i.e. IL‐6 and TNF‐*α*) upon stimulation with either Toll‐like receptor 4 (TLR4) or TLR2 ligands did not change after the intervention (Fig. S3 (https://figshare.com/s/2a0471bb2f08d5c9db56)).

**Table 2:** Effect of vancomycin on fasting leukocyte counts, LBP, and inflammatory markers.

		Preintervention	Postintervention	*P*
Leukocytes (10E9/L)	Lean	5.3 (0.8)	5.4 (1.0)	0.752
	Obese	6.0 (1.1)	6.1 (0.6)	0.718
LBP	Lean	10.44 (1.46)	11.04 (2.30)	0.258
	Obese	12.03 (2.55)	11.86 (2.61)	0.811
IL‐6 (pg/mL)	Lean	0.83 (1.82)	1.07 (1.56)	0.417
	Obese	2.03 (2.57)	2.16 (3.04)	0.720
MCP‐1 (pg/mL)	Lean	44.2 (25.5)	50.3 (21.9)	0.538
	Obese	111.5 (107.9)	91.6 (118.4)	0.361
CCR2 (MFI)	Lean	628.0 (194.2)	611.2 (149.1)	0.649
	Obese	617.2 (195.6)	518.2 (145.7)	0.029

Fasting blood leukocyte counts, plasma inflammatory markers and monocyte CCR2 expression before and after vancomycin treatment. CCR2, chemokine receptor 2; IL‐6, interleukin‐6; LBP, lipopolysaccharide‐binding protein; MCP‐1, monocyte chemoattractant protein 1. *n* = 10 per group. Data are represented as mean (SD).

### Effect of a high‐fat meal on postprandial lipids

Plasma triglycerides significantly increased after a high‐fat meal in both groups. This increase was unaffected by the intervention (Fig. S4 [https://figshare.com/s/1c7a7d643a6d9588c8ad]). Plasma HDL‐cholesterol decreased slightly but significantly after the meal. LDL‐cholesterol levels decreased by about 10% after the meal in both groups (Table S4 [https://figshare.com/s/25e137d98f1d736ab5e2]). There was no effect of vancomycin on postprandial plasma lipid concentrations (Table S4 [https://figshare.com/s/25e137d98f1d736ab5e2]).

### Postprandial translocation of LPS is unaffected by vancomycin treatment

**Figure 3:**
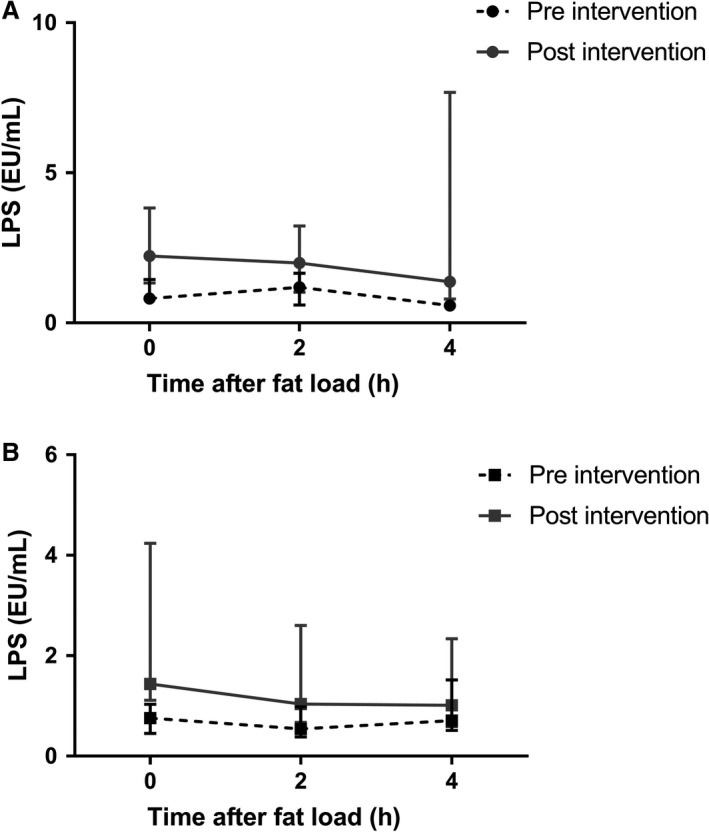
Postprandial LPS concentrations. LPS concentrations did not increase after the high‐fat meal in both the lean (A) and obese (B) group. Vancomycin treatment did not affect postprandial LPS concentrations (two‐way repeated measures ANOVA, time × treatment interaction: lean, *P* = 0.318, obese, *P* = 0.714). Lines show median and IQR.

LPS concentrations did not increase after a high‐fat meal in both groups (lean, median [IQR] at *t* = 0 h, *t* = 2 h and *t* = 4 h, respectively: 0.81 [0.63–1.45], 1.19 [0.60–1.65] and 0.58 [0.46–0.63]; Friedman test *P* = 0.016; post hoc pairwise comparisons: *t* = 0 h versus *t* = 2 h, *P* = 1.000; *t* = 0 h versus *t* = 4 h, *P* = 0.055, *t* = 2 h versus *t* = 4 h, *P* = 0.029. Obese: median [IQR] at *t* = 0 h, *t* = 2 h and *t* = 4 h, respectively: 0.76 [0.45–1.03], 0.54 [0.38–0.98] and 0.71 [0.51–1.52]; Friedman test *P* = 0.407). Although fasting LPS concentrations increased after treatment in both groups, there was no effect of vancomycin treatment on postprandial LPS concentrations (Fig. 3). LBP did not increase after the high‐fat meal, and vancomycin did not affect postprandial LBP concentrations (Table S1 [https://figshare.com/s/25e137d98f1d736ab5e2]).

### Postprandial monocyte activation is unaffected by vancomycin treatment

**Figure 4:**
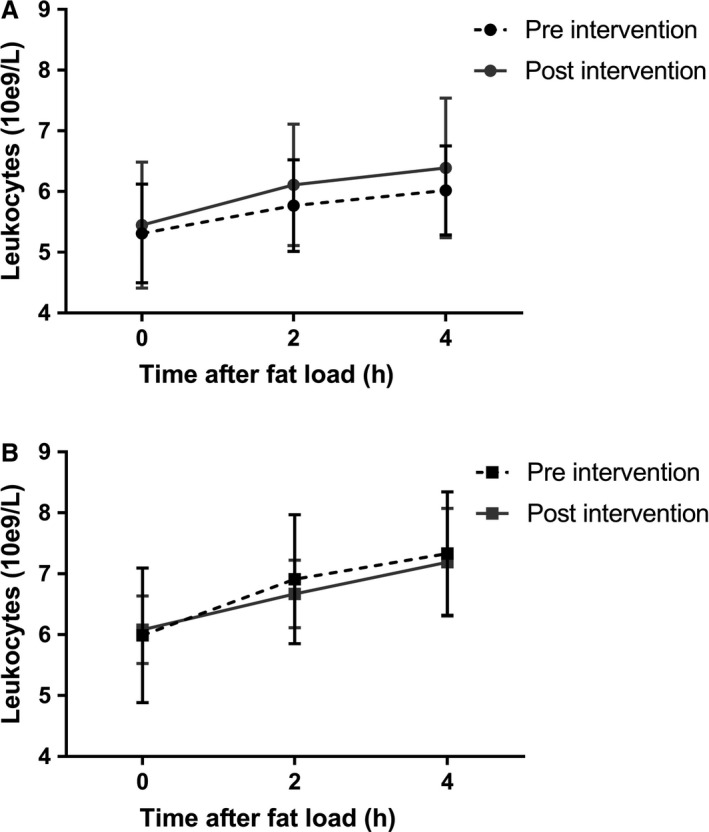
Postprandial blood leukocyte counts. Leukocyte counts significantly increased after the meal in both the lean (A) and obese (B) group (one‐way rm‐ANOVA: lean, *P* = 0.006 preintervention and *P* = 0.001 postintervention; obese, *P* = 0.001 preintervention and *P* = 0.003 postintervention). Vancomycin treatment did not affect postprandial leukocyte counts. Graphs show mean and SD.

Blood leukocyte counts significantly increased after the high‐fat meals in both groups, with no differences between pre‐ and post‐vancomycin treatment (Fig. 4 and Table S2 [https://figshare.com/s/25e137d98f1d736ab5e2]). This increase was mainly mediated by neutrophils (Table S2 [https://figshare.com/s/25e137d98f1d736ab5e2]). The increase in postprandial leukocyte counts was not accompanied by an increase in plasma IL‐6 or MCP‐1 and vancomycin did not affect the concentrations of these cytokines in the postprandial period (Table S1 [https://figshare.com/s/25e137d98f1d736ab5e2]). Distribution of monocyte types and monocyte CCR2 expression (classical, intermediate, nonclassical) also did not change after the intervention (Table S3 [https://figshare.com/s/25e137d98f1d736ab5e2]).

**Figure 5:**
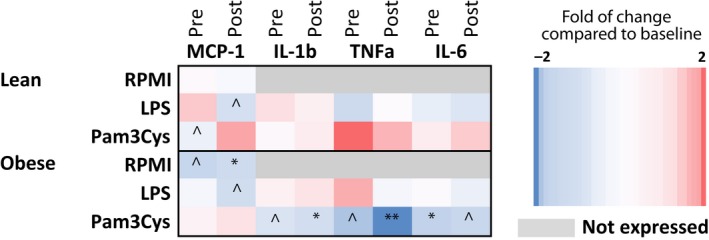
Effect of a high‐fat meal on monocyte cytokine production. Monocytes of lean and obese subjects were isolated before (*t* = 0 h) and after (*t* = 4 h) a high‐fat meal. Monocytes were stimulated with RPMI (negative control), LPS (TLR4 stimulation) or Pam3Cys (TLR2 stimulation). After 24 h, concentrations of MCP‐1, IL‐1*β*, TNF‐*α* and IL‐6 were measured in the supernatant. The heatmap shows the effects of the meal on monocyte cytokine production before (pre) and after (post) vancomycin treatment. Red indicates upregulated cytokine production after the meal compared to before the meal. Blue indicates a downregulation of cytokine production after the meal compared to before the meal. *n* = 10 per group. ^*P* < 0.1, *P < 0.05, **P < 0.01. RPMI, Roswell Park Memorial Institute.

*Ex vivo* stimulation experiments revealed that the production of several cytokines by monocytes significantly changed upon a high‐fat meal challenge and vancomycin treatment (Fig. 5) in the obese group but not in the lean group. First of all, before intervention, there was a decrease of MCP‐1 production (upon stimulation with medium only) in the obese group after a high‐fat meal. Furthermore, a high‐fat meal resulted in a non‐significant downregulation of both IL‐1*β* and TNF‐*α* upon TLR2 stimulation with Pam3Cys. Upon vancomycin treatment, these effects were even more pronounced. IL‐6 production upon TLR2 stimulation was also downregulated in the obese group, but vancomycin treatment blunted this effect. Other cytokines were not affected by either the high‐fat meal or vancomycin treatment (Fig. 5).

## Discussion

In this study, we investigated the effect of manipulation of the gut microbiota by vancomycin on the inflammatory response in the postprandial state. Despite large shifts in fecal microbiota composition with a bloom in Gram‐negative bacteria and a concomitant significant increase in fasting LPS, we did not observe a change in several postprandial inflammatory markers after a high‐fat meal in lean and obese subjects with the metabolic syndrome. This suggests that the LPS translocation may not play a large role in the short‐term postprandial response in these groups.

Previously, several groups assessed the effects of meals of varying composition on postprandial inflammation. To our knowledge, we are the first to assess the effect of manipulation of the gut microbiota on postprandial inflammation. For example, it was shown that in lean and obese subjects, a high‐fat meal consisting of bread, palm fat, salami, and boiled eggs of 1500 kcal resulted in a significant increase in plasma IL‐6 only in the obese group (Schwander et al. 2014). Another study found an average increase of plasma LPS of 50% after a high‐fat meal consisting of three slices of toast with 50 g butter in 12 healthy subjects, although there was no clear timepoint of maximum LPS concentration (Erridge et al. 2007). Deopurkar et al. (2010) reported increased plasma LPS concentrations in healthy subjects 3 h after a meal consisting of cream, but not after intake of an isocaloric glucose drink or orange juice. Studies comparing T2D patients to healthy controls found stronger increases in T2D in postprandial plasma IL‐6 (Nappo et al. 2002) and LPS (Harte et al. 2012) levels. Taken together, these studies indicate that after a high‐fat meal plasma LPS as well as inflammatory markers may increase, but they cannot confer a causal relationship between postprandial LPS translocation and low‐grade inflammation observed after acute consumption of dietary fat.

Our findings add to previous insights on the pathophysiology of the inflammatory response in the postprandial period. We observed several changes induced by a high‐fat meal in both the lean and the obese group. For example, leukocyte counts significantly increased after the meal in lean as well as in obese subjects, as was previously described (Klop et al. 2013). Moreover, the meal influenced ex vivo production of several cytokines by monocytes in the obese group. Specifically, MCP‐1 production in unstimulated monocytes and IL‐1b, TNF‐a and IL‐6 production in TLR2‐stimulated monocytes were lower 4 h after a high fat meal. We were unable to reproduce previous observations of a postprandial increase in monocyte activation (Alipour et al. 2008; Gower et al. 2011; Esser et al. 2013), although in those studies monocyte CCR2 expression was not assessed. Moreover, we did not observe an increase in plasma LPS, IL‐6 or MCP‐1 after the high‐fat meal, which is in accordance with some (Gower et al. 2011; Meher et al. 2014; Fogarty et al. 2015; Milan et al. 2017) but not other (Ghanim et al. 2009; Laugerette et al. 2011; Dijk et al. 2012) studies.

Oral vancomycin treatment significantly changed the composition of the gut microbiota as shown previously (Vrieze et al. 2014; Reijnders et al. 2016), with a relative overgrowth of potentially pathogenic Gram‐negative taxa such as Proteobacteria. Concomitantly, we found increases in fasting plasma LPS levels, possibly due to the increased presence of Gram‐negative bacteria inducing an excess of LPS in the intestinal lumen. Interestingly, *ex vivo* MCP‐1 production by monocytes that were incubated with LPS slightly decreased after vancomycin treatment only in lean fasting subjects. Similarly, a 7‐day treatment of 12 healthy subjects with ciprofloxacin, vancomycin and metronidazole was shown to lower fasting LPS‐induced TNF‐*α* production in isolated monocytes (Lankelma et al. 2016). Nevertheless, in the postprandial phase, production of cytokines by stimulated monocytes was unaffected by the intervention in our study. Moreover, both fasting and postprandial leukocyte counts, monocyte activation markers and plasma IL‐6 and MCP‐1 were unaffected by vancomycin treatment. Finally, postprandial LPS concentrations as well as LBP levels were unaffected by treatment, suggesting that manipulation of the gut microbiota did not increase postprandial bacterial translocation.

Apart from LPS measurement, quantification or sequencing of 16S rDNA in plasma is sometimes used as marker of translocation. Although this would be interesting to assess, this is not the only way to do so, nor the gold standard. Moreover, in tissues with low microbial biomass, there may be the issue of contamination (Kim et al. 2017).

LPS toxicity is related to several factors, such as bacterial origin (Schromm et al. 1998) and binding to plasma proteins (e.g. LBP, lipoproteins, CD14, endotoxin core antibodies) (Thompson et al. 2008). As LBP may be upregulated after LPS exposure in a setting of septic shock (Opal et al. 1999), it is sometimes used as a marker of LPS exposure. While LBP can augment LPS‐induced activation of monocytes by accelerating binding to CD14 at low concentration, it has inhibitory effects in high concentrations (Gutsmann et al. 2001; Zweigner et al. 2001; Thompson and Kitchens 2006). In our study we observed increased fasting plasma LPS after vancomycin intervention. However, this was not accompanied by increase in LBP. Concomitantly, no change in inflammatory markers was found.

It should be noted that we investigated the effect of a short‐term intervention (7 days) on the postprandial response to a single high‐fat meal. Although this duration is sufficient to significantly alter gut microbiota composition (Reijnders et al. 2016), it cannot be ruled out that long‐term microbiota manipulation may alter postprandial inflammatory mechanisms. The exact role of the gut microbiota in inflammation in high‐fat diets as opposed to a single high‐fat meal in humans is unclear. Previously, it was shown that in mice, a chronic high‐fat diet increased endotoxemia 2–3 fold (Cani et al. 2007). Similarly, 18 healthy subjects that were overfed (+760 kcal/day) for 8 weeks showed increased postprandial endotoxemia upon a high‐fat meal (Laugerette et al. 2014). Thus, even though we did not see an effect of the intervention on postprandial inflammation in this study, we cannot rule out a role for long‐term changes in intestinal microbiota composition or intestinal permeability in postprandial inflammation.

Our study has several strengths and weaknesses. As vancomycin treatment induced large shifts in gut microbiota composition, we were able to directly assess a potential causal role of intestinal microbes in postprandial inflammation. Moreover, our meal was high in fat content, which was reflected by a significant increase of postprandial triglycerides in both groups. Thus, our study was well suited to test the hypothesis of postprandial chylomicron‐associated LPS translocation. However, it should be noted that we were only able to assess the effect of the intervention on the fecal microbiota. As lipid uptake predominantly takes place in the small intestine, we cannot rule out that the intervention was not sufficient to increase luminal LPS concentrations and potentially alter LPS translocation in the small intestine. Of note, the increase in fasting plasma LPS could both reflect increased luminal LPS concentrations due to overgrowth of Gram‐negative bacteria or increased intestinal permeability. In this study, we did not assess intestinal permeability.

Secondly, we evaluated the postprandial inflammatory response in a lean healthy group and in an obese, metabolic syndrome group, as these groups were previously shown to have a different postprandial inflammatory response. However, as the groups had significantly different characteristics, such as age, dietary intake and baseline inflammatory parameters, we could not directly compare these two groups in this study. Instead, we assessed the effect of a vancomycin intervention in both groups separately, allowing us to do paired observations within the groups. Therefore, the study had significant power to detect differences between pre‐ and post‐intervention, despite the groups being relatively small. Nevertheless, adding a placebo‐arm would have increased the power. Thus, it would be interesting to perform a similar placebo‐controlled study in age‐matched lean and obese subjects.

In conclusion, manipulation of the gut microbiota by a short‐term course of broad‐spectrum antibiotics led to a large shift in gut microbiota composition and a significant increase in plasma LPS, but did not affect the physiology of postprandial inflammation in lean or obese subjects in this study. This suggests that LPS translocation may not drive the postprandial inflammatory response upon a single high‐fat meal.

## Endnote

At the request of the author(s), readers are herein alerted to the fact that additional materials related to raw fecal sequencing data for this manuscript may be found in the ENA repository under study PRJEB27010 at https://www.ebi.ac.uk/ena/data/view/PRJEB27010. These materials are not a part of this manuscript and have not undergone peer review by the American Physiological Society (APS). APS and the journal editors take no responsibility for these materials, for the website address, or for any links to or from it.

## Conflict of Interest

None.

## Supporting information




**Figure S1** **.** Flow cytometry gating strategy for bacteria in fecal sample. Forward and side scatter (A), SytoBC histogram with negative control (no stain) (B), example of stained bacteria from a pre‐vancomycin fecal sample (C) and example from a post‐vancomycin fecal sample (D).

**Figure S2** **.** Flow cytometry gating strategy for monocytes and their subtypes in a PBMC sample. Leukocytes were gated using forward and side scatter (A), monocytes were selected using CD14, CD16 and HLA‐DR (B and C) and finally the monocyte subtypes were gated. We distinguished classical (CD14++ CD16‐), intermediate (CD14++, CD16+) and non‐ classical (CD14+ CD16+) monocytes (D).

**Figure S3** **.** Monocytes of fasting lean and obese subjects were isolated and stimulated with RPMI (negative control), LPS (TLR4 stimulation) or Pam3Cys (TLR2 stimulation). After 24h, concentrations of MCP‐1, IL‐1*β*, TNF‐*α* and IL‐6 were measured in the supernatant. The heatmap shows the effects of vancomycin on monocyte cytokine production. Red indicates upregulated cytokine production after treatment compared to before treatment. Blue indicates a downregulation of cytokine production after treatment compared to before treatment. n=10 per group. ^p < 0.1, *p < 0.05.

**Figure S4** **.** Triglycerides significantly increased after the meal in both the lean (A) and obese (B) group (oneway repeated measurements‐ANOVA: lean, p < 0.001 both pre‐ and post‐intervention; obese, p < 0.001 both pre‐ and post‐intervention). There was no effect of vancomycin on postprandial triglyceride concentrations (two‐way repeated measurements‐ANOVA, time * treatment interaction: lean, p = 0.436, obese, p = 0.483). Graphs show mean and SD.

**Table S1** **.** Plasma LBP and cytokines fasting at fasting, 2 and 4 hours after an oral fatload before (pre) and after (post) vancomycin treatment. IL‐6, Interleukin‐6. LBP, lipopolysaccharde‐binding protein; MCP‐1, monocyte chemoattractant protein 1; p meal represents differences between t = 0h, t = 2h, and t = 4h (one‐way repeated measurements‐ANOVA). p intervention represents the overall intervention effect (two‐way rm‐ANOVA, time * treatment interaction). Data are mean (SD).
**Table S2** **.** Blood differentiated leukocyte counts at fasting, 2 and 4 hours after an oral fatload before (pre) and after (post) vancomycin treatment. p meal represents differences between t = 0h, t = 2h, and t = 4h (oneway rm‐ANOVA). p intervention represents the overall intervention effect (two‐way rm‐ANOVA, time *treatment interaction). Data are mean (SD).
**Table S3** **.** Monocyte type distribution based on flow cytometry at fasting and 4 hours after an oral fatload before (pre) and after (post) vancomycin treatment. Monocytes were categorized as type 1 (classical), type 2 (intermediate) or type 3 (non‐classical) based on CD14/CD16 expression (type 1, CD14++ CD16−; type 2, CD14++CD16+; type 3, CD14+CD16+). CCR2, C‐C chemokine receptor type 2. p meal represents differences between t = 0h and t = 4h (paired t‐test). p intervention represents difference in delta (t = 4h minus t = 0h) between pre and post intervention (paired t‐test). Data are mean (SD).
**Table S4** **.** Plasma concentrations of lipids fasting and 2 and 4 hours after an oral fat lipoprotein cholesterol; LDL‐c, low‐density lipoprotein cholesterol; n, number of patients. p meal represents differences between t = 0h, t = 2h, and t = 4h (one‐ way repeated measurement‐ANOVA). p intervention represents the overall intervention effect (two‐way repeated measurement‐ANOVA, time * treatment interaction). Data are mean (SD).
