# Supplementary figures and images for: Oral vancomycin treatment does not alter markers of postprandial inflammation in lean and obese subjects
Source: Physiol Rep. 2019 Aug 18;7(16):e14199. doi: 10.14814/phy2.14199 (PMC6698488; doi:10.14814/phy2.14199)

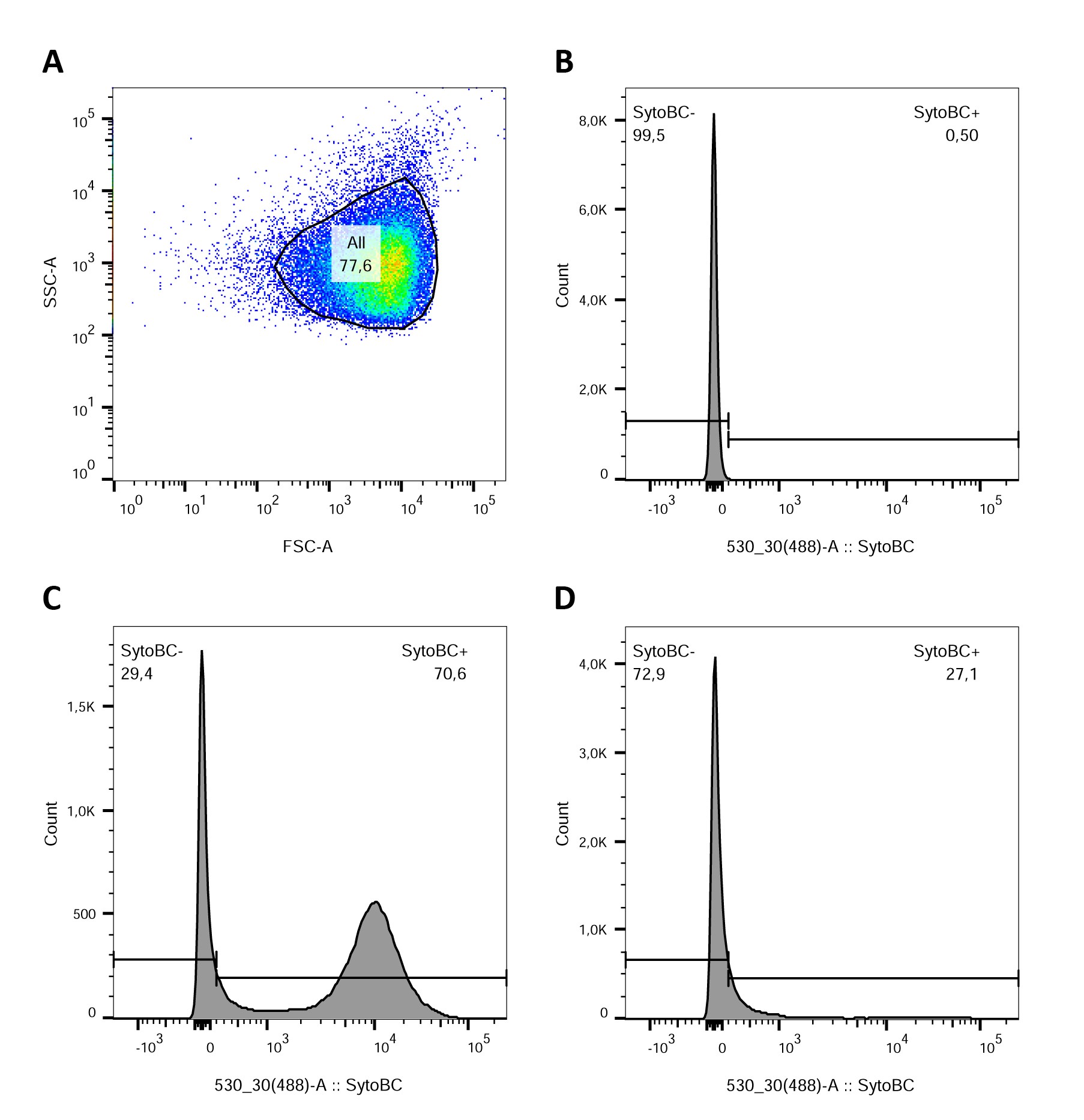

Supplement: Supplementary file 1 — Figure S1 . Flow cytometry gating strategy for bacteria in fecal sample. Forward and side scatter (A), SytoBC histogram with negative control (no stain) (B), example of stained bacteria from a pre‐vancomycin fecal sample (C) and example from a post‐vancomycin fecal sample (D). [file PHY2-7-e14199-s001.jpg]

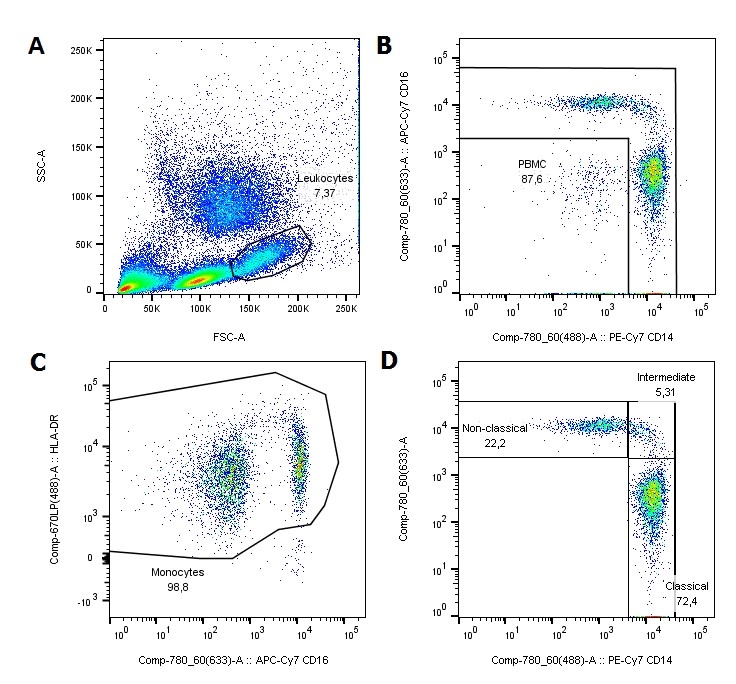

Supplement: Supplementary file 2 — Figure S2 . Flow cytometry gating strategy for monocytes and their subtypes in a PBMC sample. Leukocytes were gated using forward and side scatter (A), monocytes were selected using CD14, CD16 and HLA‐DR (B and C) and finally the monocyte subtypes were gated. We distinguished classical (CD14++ CD16‐), intermediate (CD14++, CD16+) and non‐ classical (CD14+ CD16+) monocytes (D). [file PHY2-7-e14199-s002.jpg]

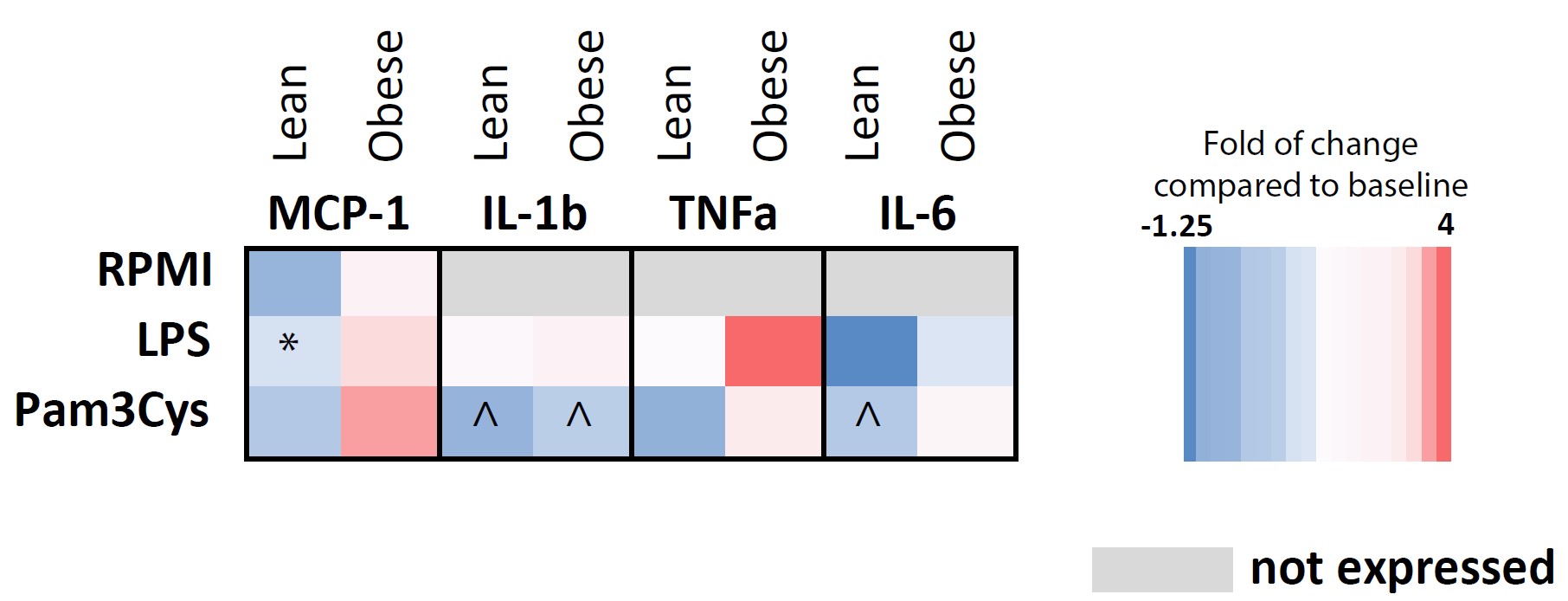

Supplement: Supplementary file 3 — Figure S3 . Monocytes of fasting lean and obese subjects were isolated and stimulated with RPMI (negative control), LPS (TLR4 stimulation) or Pam3Cys (TLR2 stimulation). After 24h, concentrations of MCP‐1, IL‐1β, TNF‐α and IL‐6 were measured in the supernatant. The heatmap shows the effects of vancomycin on monocyte cytokine production. Red indicates upregulated cytokine production after treatment compared to before treatment. Blue indicates a downregulation of cytokine production after treatment compared to before treatment. n=10 per group. ^p < 0.1, *p < 0.05. [file PHY2-7-e14199-s003.jpg]

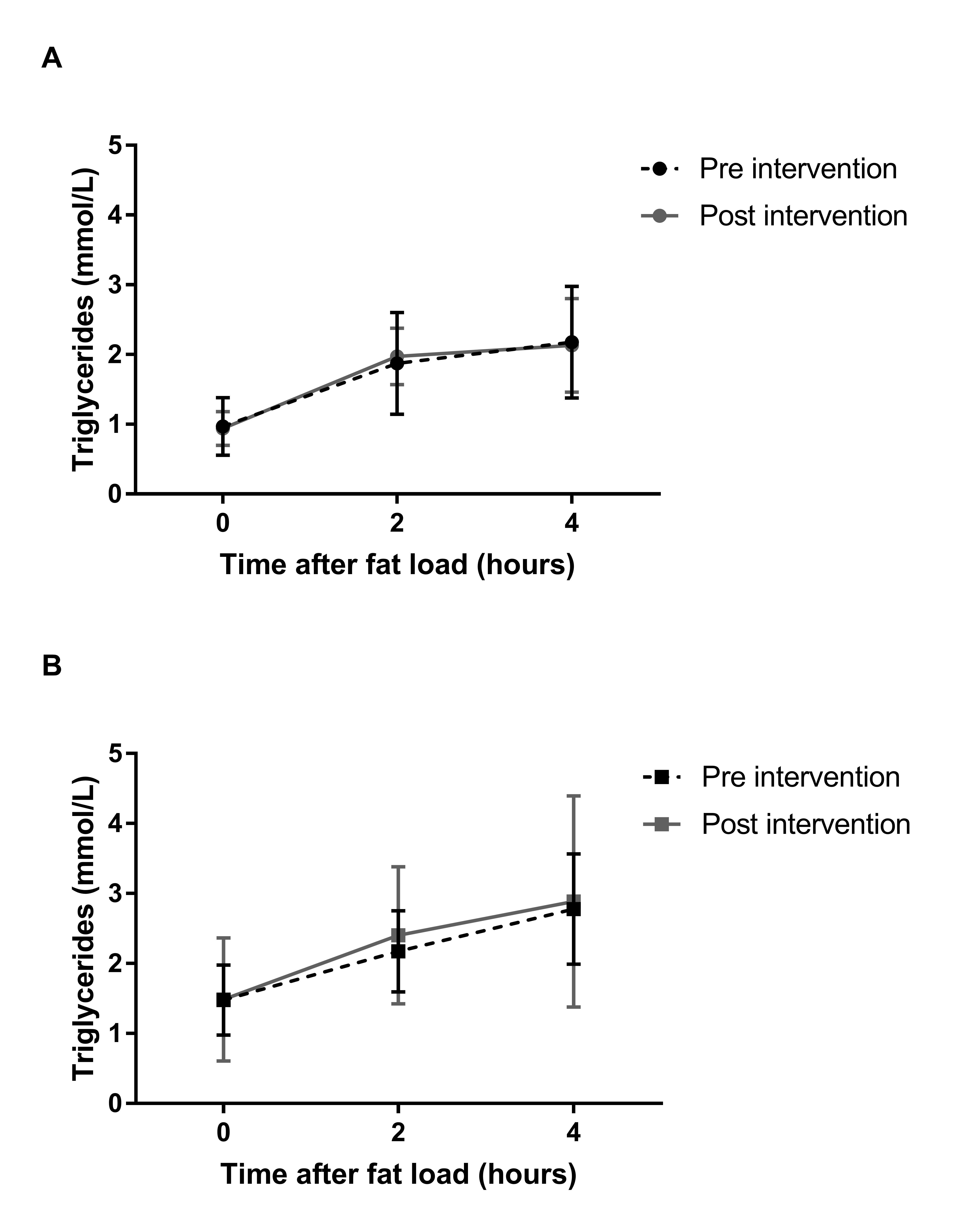

Supplement: Supplementary file 4 — Figure S4 . Triglycerides significantly increased after the meal in both the lean (A) and obese (B) group (oneway repeated measurements‐ANOVA: lean, p < 0.001 both pre‐ and post‐intervention; obese, p < 0.001 both pre‐ and post‐intervention). There was no effect of vancomycin on postprandial triglyceride concentrations (two‐way repeated measurements‐ANOVA, time * treatment interaction: lean, p = 0.436, obese, p = 0.483). Graphs show mean and SD. [file PHY2-7-e14199-s004.jpg]
