# Supplementary material for: Oral vancomycin treatment does not alter markers of postprandial inflammation in lean and obese subjects
Source: Physiol Rep. 2019 Aug 18;7(16):e14199. doi: 10.14814/phy2.14199 (PMC6698488; doi:10.14814/phy2.14199)
Supplement: Supplementary file 5 — Table S1 . Plasma LBP and cytokines fasting at fasting, 2 and 4 hours after an oral fatload before (pre) and after (post) vancomycin treatment. IL‐6, Interleukin‐6. LBP, lipopolysaccharde‐binding protein; MCP‐1, monocyte chemoattractant protein 1; p meal represents differences between t = 0h, t = 2h, and t = 4h (one‐way repeated measurements‐ANOVA). p intervention represents the overall intervention effect (two‐way rm‐ANOVA, time * treatment interaction). Data are mean (SD). Table S2 . Blood differentiated leukocyte counts at fasting, 2 and 4 hours after an oral fatload before (pre) and after (post) vancomycin treatment. p meal represents differences between t = 0h, t = 2h, and t = 4h (oneway rm‐ANOVA). p intervention represents the overall intervention effect (two‐way rm‐ANOVA, time *treatment interaction). Data are mean (SD). Table S3 . Monocyte type distribution based on flow cytometry at fasting and 4 hours after an oral fatload before (pre) and after (post) vancomycin treatment. Monocytes were categorized as type 1 (classical), type 2 (intermediate) or type 3 (non‐classical) based on CD14/CD16 expression (type 1, CD14++ CD16−; type 2, CD14++CD16+; type 3, CD14+CD16+). CCR2, C‐C chemokine receptor type 2. p meal represents differences between t = 0h and t = 4h (paired t‐test). p intervention represents difference in delta (t = 4h minus t = 0h) between pre and post intervention (paired t‐test). Data are mean (SD). Table S4 . Plasma concentrations of lipids fasting and 2 and 4 hours after an oral fat lipoprotein cholesterol; LDL‐c, low‐density lipoprotein cholesterol; n, number of patients. p meal represents differences between t = 0h, t = 2h, and t = 4h (one‐ way repeated measurement‐ANOVA). p intervention represents the overall intervention effect (two‐way repeated measurement‐ANOVA, time * treatment interaction). Data are mean (SD). [file PHY2-7-e14199-s005.doc]

**Supplementary Materials**

*Gut microbiota DNA isolation and 16S sequencing*

Total genomic DNA was isolated from 250 mg of feces using an adapted repeated bead beating method based on a protocol previously described (3). Fecal samples were placed in bead-beating tubes and extracted twice in lysis buffer (STARbuffer, Roche, Basel, Switzerland) with bead-beating at 5.5 m/s for 60 s, 3 times in 20 s intervals in a Precellys 24 (Bertin). After each bead-beating cycle, samples were heated to 95ºC for 15 min and then centrifuged at full speed for 5 min at 4ºC. Supernatants from the two extractions were pooled and purified with the Maxwell RSC Blood DNA kit (Promega, Madison, Wisconsin, U.S.A.). Amplicons containing the barcoded V4 region of 16S genes were amplified using an adapted one-step PCR method (2). Forward Index Primer (Illumina Adapter, Index, pad, link, 16Sf) and Reverse Index Primer (Illumina Adapter, Index, pad, link, 16Sr) were used (1).

Twenty ng of template DNA was used in the PCR mix: 6 μL 5× HF buffer (Thermo Fisher Scientific, Waltham, Massachusetts, U.S.A.), 0.75 μL PCR Grade Nucleotide Mix (10 μM) (Thermo Fisher Scientific, Waltham, Massachusetts, U.S.A.), 0.3 μL Phusion DNA Polymerase (2 U/µL), 18.95 μL nuclease free water, 1.5 μL Forward Index Primer (10 μM) and 1.5 μL Reverse Index Primer (10 μM). The amplification program: initial denaturation at 98°C for 30 s; 25 cycles of denaturation at 98°C for 10 s, annealing at 55°C for 20 s, elongation at 72°C for 90 s; extension at 72°C for 10 min (1). PCR was performed using a Biometra Thermocycler (Westburg, Leusden, The Netherlands). To confirm the presence of the PCR product, the amplification product was tested on a 1% agarose gel. PCR products were purified using the Biomek FX robot (Beckman Coulter, Brea, California, U.S.A.) with Ampure XP beads (Beckman Coulter, Brea, California, U.S.A.). Amplicons were quantified using the Qubit® dsDNA BR Assay Kit (Thermo Fisher Scientific, Waltham, Massachusetts, U.S.A.). The samples, each labeled with a unique index, were equal molar pooled. The quality and quantity of the pooled library was checked using a High Sensitive DNA chip (Agilent, Santa Clara, California, U.S.A.) on the Bioanalyzer 21000 (Agilent, Santa Clara, California, U.S.A.) and Qubit® dsDNA BR Assay Kit. The Pooled library was sequenced by Illumina Miseq (V3,600) sequencing (1).

| **Supplemental Table 1.** | | | | | | | | |  | | |  | |  | | |  | | |  |
| --- | --- | --- | --- | --- | --- | --- | --- | --- | --- | --- | --- | --- | --- | --- | --- | --- | --- | --- | --- | --- |
|  |  |  | **Lean (n = 10)** | |  |  |  | | | **Obese (n=10)** | | | | |  | | |  | | |
|  |  | **t = 0** | **t = 2** | **t = 4** | ***p* meal** | ***p* inter-vention** | | **t = 0** | | | **t = 2** | | **t = 4** | | | ***p* meal** | | | ***p* inter-vention** | |
| LBP | Pre | 10.44 (1.46) | 10.55 (1.23) | 10.99 (1.50) | 0.096 | 0.128 | | 12.03 (2.55) | | | 11.79 (1.97) | | 11.86 (1.94) | | | 0.623 | | | 0.332 | |
|  | Post | 11.04 (2.30) | 10.93 (1.58) | 10.76 (1.58) | 0.556 |  | | 11.86 (2.61) | | | 11.39 (2.37) | | 11.87 (2.50) | | | 0.037 | | |  | |
| IL-6 | Pre | 0.83 (1.82) | 1.15 (2.08) | 0.95 (1.76) | 0.153 | 0.347 | | 2.03 (2.57) | | | 3.20 (5.67) | | 2.66 (3.84) | | | 0.417 | | | 0.339 | |
|  | Post | 1.07 (1.56) | 0.80 (1.46) | 0.87 (1.46) | 0.496 |  | | 2.16 (3.04) | | | 1.96 (3.04) | | 2.21 (3.34) | | | 0.347 | | |  | |
| MCP-1 | Pre | 44.2 (25.5) | 32.0 (27.5) | 42.4 (15.9) | 0.053 | 0.084 | | 111.5 (107.9) | | | 69.5 (101.6) | | 69.6 (94.6) | | | 0.013 | | | 0.488 | |
|  | Post | 50.3 (21.9) | 40.9 (22.6) | 58.7 (26.5) | 0.037 |  | | 91.6 (118.4) | | | 73.4 (76.7) | | 91.3 (100.0) | | | 0.286 | | |  | |

| **Supplemental Table 2.** | | | | | | |  |  | |  |  |
| --- | --- | --- | --- | --- | --- | --- | --- | --- | --- | --- | --- |
|  |  |  | **Lean (n = 10)** | |  |  |  | **Obese (n=10)** | |  |  |
|  |  | **t = 0** | **t = 2** | **t = 4** | ***p* meal** | ***p* inter-vention** | **t = 0** | **t = 2** | **t = 4** | ***p* meal** | ***p* inter-vention** |
| Leukocytes (10E9/L) | Pre | 5.3 (0.8) | 5.8 (0.8) | 6.0 (0.7) | 0.006 | 0.478 | 6.0 (1.1) | 6.9 (1.0) | 7.3 (1.0) | 0.001 | 0.753 |
|  | Post | 5.4 (1.0) | 6.1 (1.0) | 6.4 (1.1) | 0.001 |  | 6.1 (0.6) | 6.7 (0.6) | 7.2 (0.9) | 0.003 |  |
| Neutrophils (10E9/L) | Pre | 2.7 (0.7) | 3.3 (0.8) | 3.5 (0.9) | <0.001 | 0.886 | 3.5 (1.1) | 4.3 (1.2) | 4.4 (0.8) | 0.008 | 0.543 |
|  | Post | 3.0 (0.9) | 3.6 (1.0) | 3.7 (1.0) | 0.003 |  | 3.5 (0.7) | 4.0 (0.7) | 4.1 (0.7) | <0.001 |  |
| Lymphocytes (10E9/L) | Pre | 1.9 (0.4) | 1.8 (0.4) | 1.9 (0.4) | 0.233 | 0.104 | 1.8 (0.4) | 1.9 (0.5) | 2.2 (0.6) | 0.010 | 0.825 |
|  | Post | 1.7 (0.3) | 1.8 (0.3) | 1.9 (0.3) | 0.095 |  | 1.7 (0.5) | 1.9 (0.5) | 2.2 (0.7) | <0.001 |  |
| Monocytes (10E9/L) | Pre | 0.5 (0.1) | 0.5 (0.1) | 0.5 (0.1) | 0.748 | 0.383 | 0.5 (0.2) | 0.5 (0.2) | 0.6 (0.2) | 0.168 | 0.267 |
|  | Post | 0.5 (0.1) | 0.5 (0.1) | 0.5 (0.1) | 0.227 |  | 0.5 (0.1) | 0.5 (0.1) | 0.6 (0.1) | 0.016 |  |

| **Supplemental Table 3.** | | | | | |  |  |  |  | |  |
| --- | --- | --- | --- | --- | --- | --- | --- | --- | --- | --- | --- |
|  |  | **Lean (n = 10)** | |  |  | **Obese (n=10)** | |  |  | |  |
|  |  | **t = 0** | **t = 4** | ***p* meal** | ***p* inter-vention** | **t = 0** | **t = 4** | ***p* meal** | | ***p* inter-vention** | |
| Monocyte type 1 (%) | Pre | 89.9 (3.7) | 88.9 (3.6) | 0.154 | 0.633 | 85.8 (6.2) | 86.4 (5.2) | 0.313 | | 0.778 | |
|  | Post | 91.2 (3.9) | 90.4 (3.4) | 0.304 |  | 84.3 (9.0) | 85.6 (4.0) | 0.695 | |  | |
| Monocyte type 2 (%) | Pre | 2.5 (0.8) | 2.6 (0.6) | 0.778 | 0.185 | 3.7 (1.4) | 3.1 (1.4) | 0.006 | | 0.064 | |
|  | Post | 2.4 (1.2) | 2.7 (1.0) | 0.186 |  | 3.6 (1.6) | 3.3 (1.7) | 0.278 | |  | |
| Monocyte type 3 (%) | Pre | 7.2 (3.0) | 8.1 (2.9) | 0.159 | 0.225 | 10.2 (5.6) | 10.3 (4.8) | 0.804 | | 0.203 | |
|  | Post | 6.2 (3.0) | 6.6 (2.5) | 0.456 |  | 9.2 (4.7) | 10.8 (3.8) | 0.134 | |  | |
| Monocyte CCR2 expression (AU) | Pre | 628 (194) | 667 (184) | 0.187 | 0.543 | 617 (196) | 708 (190) | 0.014 | | 0.124 | |
|  | Post | 611 (149) | 668 (119) | 0.109 |  | 518 (146) | 543 (143) | 0.262 | |  | |

| **Supplemental Table 4.** | | | | | | |  |  |  |  |  |  |  |  |  |
| --- | --- | --- | --- | --- | --- | --- | --- | --- | --- | --- | --- | --- | --- | --- | --- |
|  |  |  | **Lean (n = 10)** | |  |  |  | **Obese (n=10)** | |  |  |  | | | |
|  |  | **t = 0** | **t = 2** | **t = 4** | ***p* meal** | ***p* inter-vention** | **t = 0** | **t = 2** | **t = 4** | ***p* meal** | ***p* inter-vention** |  | | | |
| Total cholesterol (mmol/L) | Pre | 4.19 (0.59) | 4.37 (0.63) | 4.41 (0.66) | <0.001 | 0.663 | 5.49 (0.84) | 5.62 (0.94) | 5.64 (0.89) | 0.060 | 0.575 |  | | | |
|  | Post | 4.34 (0.72) | 4.53 (0.73) | 4.53 (0.73) | <0.001 |  | 5.38 (0.82) | 5.56 (0.90) | 5.62 (0.90) | 0.009 |  |  | | | |
| HDL-c (mmol/L) | Pre | 1.23 (0.26) | 1.30 (0.28) | 1.19 (0.25) | 0.003 | 0.658 | 1.25 (0.18) | 1.24 (0.21) | 1.15 (0.21) | <0.001 | 0.407 |  | | | |
|  | Post | 1.27 (0.22) | 1.36 (0.27) | 1.21 (0.27) | 0.001 |  | 1.19 (0.22) | 1.24 (0.16) | 1.13 (0.28) | 0.047 |  |  | | | |
| LDL-c (mmol/L) | Pre | 2.51 (0.46) | 2.21 (0.49) | 2.23 (0.49) | <0.001 | 0.490 | 3.56 (0.91) | 3.39 (0.90) | 3.22 (0.80) | 0.005 | 0.419 |  | | | |
|  | Post | 2.64 (0.58) | 2.26 (0.53) | 2.34 (0.55) | <0.001 |  | 3.50 (0.89) | 3.23 (0.98) | 3.17 (1.00) | 0.002 |  |  | | | |
| Triglycerides (mmol/L) | Pre | 0.97 (0.41) | 1.87 (0.73) | 2.18 (0.80) | <0.001 | 0.436 | 1.48 (0.50) | 2.17 (0.58) | 2.78 (0.79) | <0.001 | 0.483 |  | | | |
|  | Post | 0.96 (0.24) | 1.97 (0.41) | 2.13 (0.67) | <0.001 |  | 1.49 (0.88) | 2.40 (0.98) | 2.88 (1.51) | <0.001 |  |  | | | |
| Apo A1 (mg/dL) | Pre | 148 (20) | 153 (22) | 154 (22) | 0.013 | 0.732 | 149 (22) | 148 (19) | 149 (22) | 0.723 | 0.123 |  | | | |
|  | Post | 153 (20) | 159 (23) | 159 (23) | 0.023 |  | 146 (21) | 150 (19) | 151 (20) | 0.013 |  |  | | | |
| Apo B (mg/dL) | Pre | 67.0 (13.4) | 69.5 (14.2) | 68.9 (14.4) | 0.002 | 0.899 | 104 (19) | 105 (22) | 105 (21) | 0.533 | 0.622 |  | | | |
|  | Post | 68.5 (14.1) | 71.1 (14.5) | 70.8 (14.6) | 0.004 |  | 101 (21) | 103 (22) | 104 (22) | 0.083 |  |  | | | |

**References**

1. **Kozich JJ, Westcott SL, Baxter NT, Highlander SK, and Schloss PD.** Development of a dual-index sequencing strategy and curation pipeline for analyzing amplicon sequence data on the MiSeq Illumina sequencing platform. *Appl Environ Microbiol* 79: 5112-5120, 2013.

2. **Ramiro-Garcia JH, G.D.A.; Giatsis, C.; Sipkema, D.; Zoetendal, E.G.; Schaap, P.J.; Smidt, H.** NG-tax, a highly accurate and validated pipeline for analysis of 16S rRNA amplicons from complex biomes [version 1; referees: awaiting peer review]. *F1000Research* 5: 1791, 2016.

3. **Salonen A, Nikkila J, Jalanka-Tuovinen J, Immonen O, Rajilic-Stojanovic M, Kekkonen RA, Palva A, and de Vos WM.** Comparative analysis of fecal DNA extraction methods with phylogenetic microarray: effective recovery of bacterial and archaeal DNA using mechanical cell lysis. *J Microbiol Methods* 81: 127-134, 2010.

**Table and Figure legends**

**Supplemental Table 1**

Title: Effect of vancomycin on plasma LBP and cytokines

Legend: Plasma LBP and cytokines fasting and 2 and 4 hours after an oral fat load before (pre) and after (post) vancomycin treatment. IL-6, interleukin-6; LBP, lipopolysaccharde-binding protein; MCP-1, monocyte chemoattractant protein 1; *p* meal represents differences between t=0h, t=2h, and t=4h (onw-way rm-ANOVA). *p* intervention represents the overall intervention effect (two-way rm-ANOVA, time * treatment interaction). Data are mean (SD).

**Supplemental Table 2**

Title: Effect of vancomycin on blood leukocyte counts

Legend: Blood differentiated leukocyte counts fasting and 2 and 4 hours after an oral fat load before (pre) and after (post) vancomycin treatment. *p* meal represents differences between t=0h, t=2h, and t=4h (one-way rm-ANOVA). *p* intervention represents the overall intervention effect (two-way rm-ANOVA, time * treatment interaction). Data are mean (SD).

**Supplemental Table 3**

Title: Effect of vancomycin on monocytes

Legend: Monocyte type distribution based on flow cytometry fasting and 4 hours after an oral fat load before (pre) and after (post) vancomycin treatment. Monocytes were categorized as type 1 (classical), type 2 (intermediate) or type 3 (non-classical) based on CD14/CD16 expression (type 1, CD14++ CD16−; type 2, CD14++CD16+; type 3, CD14+CD16+). CCR2, C-C chemokine receptor type 2. *p* meal represents differences between t=0h and t=4h (paired t-test). *p* intervention represents difference in delta (t=4h minus t=0h) between pre and post intervention (paired t-test). Data are mean (SD).

**Supplemental Table 4**

Title: Postprandial plasma lipids

Legend: Plasma concentrations of lipids fasting and 2 and 4 hours after an oral fat load before (pre) and after (post) vancomycin treatment. HDL-c, high density lipoprotein cholesterol; LDL-c, low-density lipoprotein cholesterol; n, number of patients. p meal represents differences between t=0h, t=2h, and t=4h (one-way rm-ANOVA). p intervention represents the overall intervention effect (two-way rm-ANOVA, time * treatment interaction). Data are mean (SD).

**Supplemental Figure 1**

Title: Gating strategy for SytoBC staining of fecal bacteria

Legend: Flow cytometry gating strategy for bacteria in fecal sample. Forward and side scatter (A), SytoBC histogram with negative control (no stain) (B), example of stained bacteria from a pre-vancomycin fecal sample (C) and example from a post-vancomycin fecal sample (D).

**Supplemental Figure 2**

Title: Gating strategy for flow cytometry of monocytes

Legend: Flow cytometry gating strategy for monocytes and their subtypes in a PBMC sample. Leukocytes were gated using forward and side scatter (A), monocytes were selected using CD14, CD16 and HLA-DR (B and C) and finally the monocyte subtypes were gated. We distinguished classical (CD14++ CD16-), intermediate (CD14++, CD16+) and non-classical (CD14+ CD16+) monocytes (D).

**Supplemental Figure 3**

Title: Effect of vancomycin treatment on fasting monocyte cytokine production

Legend: Monocytes of fasting lean and obese subjects were isolated and stimulated with RPMI (negative control), LPS (TLR4 stimulation) or Pam3Cys (TLR2 stimulation). After 24h, concentrations of MCP-1, IL-1β, TNF-α and IL-6 were measured in the supernatant. The heatmap shows the effects of vancomycin on monocyte cytokine production. Red indicates upregulated cytokine production after treatment compared to before treatment. Blue indicates a downregulation of cytokine production after treatment compared to before treatment. n=10 per group. ^*p*<0.1, **p*<0.05.

**Supplemental Figure 4**

Title: Postprandial triglycerides

Legend: Triglycerides significantly increased after the meal in both the lean (A) and obese (B) group (one-way rm-ANOVA: lean, p<0.001 both pre- and post-intervention; obese, p<0.001 both pre- and post-intervention). There was no effect of vancomycin on postprandial triglyceride concentrations (two-way rm-ANOVA, time * treatment interaction: lean, p=0.436, obese, p=0.483). Graphs show mean and SD.
